# An Examination System to Detect Deep Vein Thrombosis of a Lower Limb Using Light Reflection Rheography

**DOI:** 10.3390/s21072446

**Published:** 2021-04-02

**Authors:** Shing-Hong Liu, Jia-Jung Wang, Wenxi Chen, Kuo-Li Pan, Chun-Hung Su

**Affiliations:** 1 Department of Computer Science and Information Engineering, Chaoyang University of Technology, Taichung 413310, Taiwan; shliu@cyut.edu.tw; 2 Department of Biomedical Engineering, I-Shou University, Kaohsiung 84001, Taiwan; 3 Biomedical Information Engineering Laboratory, The University of Aizu, Aizu-Wakamatsu City 965-8580, Fukushima, Japan; wenxi@u-aizu.ac.jp; 4 Division of Cardiology, Chiayi Chang Gung Memorial Hospital, Chiayi City 61363, Taiwan; 5 Department of Traditional Chinese Medicine, College of Medicine, Chang Gung University, Taoyuan City 33305, Taiwan; 6 Institute of Medicine, School of Medicine, Chung-Shan Medical University, Taichung 40201, Taiwan; such197408@gmail.com; 7 Department of Internal Medicine, Chung-Shan Medical University Hospital, Taichung 40201, Taiwan

**Keywords:** deep vein thrombosis, light reflection rheography, wearable device, photoplethysmography

## Abstract

Deep vein thrombosis (DVT) of lower limbs can easily arise from prolonged sitting or standing. Elders and pregnant women are most likely to have this disease. When the embolus of DVT comes to pass the lung, it will become a life-threatening disease. Thus, for DVT disease, early detection and the early treatment are needed. The goal of this study was to develop an examination system to be used at non-medical places to detect the DVT of lower limbs with light reflection rheography (LRR). Consisting of a wearable device and a mobile application (APP), the system is operated in a wireless manner to control the actions of sensors and display and store the LRR signals on the APP. Then, the recorded LRR signals are processed to find the parameters of DVT examination. Twenty subjects were recruited to perform experiments. The veins of lower limbs were occluded by pressuring the cuff up to 100 mmHg and 150 mmHg to simulate the slight and serious DVT scenarios, respectively. Six characteristic parameters were defined to classify whether there was positive or negative DVT using the receiver operating characteristic curves, including the slopes of emptying and refilling curves in the LRR signal, and the changes of venous pump volume. Under the slight DVT scenario (0 mmHg vs. 100 mmHg), the first three parameters, m_10_, m_40_, and m_50_, had accuracies of 72%, 69%, and 69%, respectively. Under the serious DVT scenario (0 mmHg vs. 150 mmHg), m_10_, m_40_, and m_50_ achieved accuracies of 73%, 76%, and 73%, respectively. The experimental results show that this proposed examination system may be practical as an auxiliary tool to screen DVT in homecare settings.

## 1. Introduction

Deep vein thrombosis (DVT) occurs when a blood clot forms in one or more of the deep veins in the human body, which will change the blood flow of veins and result in poor blood circulation. Detection of DVT is usually a clinical challenge for doctors of all medical specialties, and deterioration of such disease may make the treatment process more complicated. Moreover, DVT can happen even in the absence of serious disorders. As investigated, thrombosis can occur in any part of the venous system, but it appears most frequently at the deep veins of the legs [1]. Post-thrombotic syndromes arising from long-term morbidity can be common and substantial. Embolization of thrombus occurring in the lung due to DVT can be fatal. Furthermore, DVT is highly prevalent in Taiwan, and its treatment consumes a lot of health insurance funds [2]. Thus, both early screening and proper treatment of DVT are important to prevent it from worsening and happening again.

Traveler’s thrombosis is caused by sitting still in a transport vehicle for a long time, and blood clots are formed and deposited inside the deep veins of lower limbs, which constitutes DVT [3]. Scurr et al. have indicated incidences of 10% of asymptomatic calf vein thrombosis in air travelers above 50 years of age and traveling for more than 8 h [4]. Hence, the main risk factors of DVT include prolonged sitting or standing, family history of thrombus, pregnancy, diabetes, hyperlipidemia, and hypertension. The venous thrombus could flow to the pulmonary vein to cause the pulmonary embolism. Therefore, the early diagnosis and treatment of DVT can prevent the pulmonary embolism.

Clinical examinations of DVT are currently performed using ultrasound or venography, and they all need to be carried out by professional technicians or physicians in a hospital [5,6]. Moreover, these examinations are not performed in general clinics. In 1976, Hull et al. proposed impedance plethysmography [7,8] to detect DVT. Sandler et al. have examined DVT with Doppler ultrasound, impedance plethysmography, and venoscan, and the accuracy rates were 65%, 80%, and 82%, respectively, by comparison with the X-ray venography as the standard [9]. Thus, these techniques all have some limitations for the diagnoses of DVT, except for venography. Ginsberg et al. have applied D-dimer testing and impedance plethysmography to examine 98 patients with DVT symptoms. Their results showed that, when both methods were tested as “negative” after detailed examination, the accuracy rate was as high as 98% [10]. This is enough to show that the vein plethysmography could be an auxiliary tool to examine DVT.

Although the impedance plethysmography has good sensitivity in detecting venous thrombosis in the hip, thigh, and popliteal area, it is not very sensitive to calf vein thrombosis [8,11]. The study by Goodacre et al. found that the most common location for venous thrombosis is in about 50% of the lower limbs [12]. In 1984, Shepard et al. published a technique using light reflection rheography (LRR) to examine DVT in the calf [13]. Some studies for the applications of LRR were published in 1992 [14] and 1993 [15]. The LRR uses reflective photoplethysmography (PPG) to detect the blood volume changes in the superficial vein. According to the studies of Thomas et al. [16], LRR has a sensitivity of 92% and a specificity of 84% in detecting acute thrombosis of the calf. This technique has a negative predictive value of 92% in selecting those patients with no thrombosis. Thus, LRR can be employed to develop a wearable device to prevent the varicose veins of lower limbs.

In recent years, people have been fond of using wearable devices to monitor their bodies’ condition in daily life. In various wearable devices, an exercise watch or band can be utilized to measure the heart rate and the number of steps [17,18], an electrocardiogram (ECG) patch can be used to record a long-term ECG signal [19], and an electromyogram (EMG) patch can be applied to detect muscle fatigue in real time [20]. The major differences between the wearable devices and medical apparatus include the size, weight, and usability. Thus, it is now possible for users to make use of these wearable devices to take care of their health conditions in daily life. These physiological data can be further utilized by artificial intelligent technologies to predict or diagnose diseases [21,22]. However, how to design wearable devices having the same functions as an apparatus with medical grade is a challenge to engineers, because such medical apparatus is usually large and heavy. Now, the advantages of advanced sensor chips are leading to lower power consumption, smaller size, and digitization in devices such as ECG sensors [23], PPG sensors [24], and accelerometers [25]. With those advanced sensor chips, it becomes more possible to develop a medical-grade wearable device.

LRR, a noninvasive and non-ionizing radiation measurement technique, can be applied to observe whether a lower limb has DVT by means of reflective PPG sensors. In the examination procedure, the physician has to look for an appropriate position for placing the PPG sensor to obtain a reliable PPG signal, and guides the patient to perform the dorsiflexion at the ankle to push the blood in the vein of the calf back to the heart. However, it is hard for users, at home, to check the vein condition by such medical grade apparatus. The aim of this study is to develop an examination system, available for use in non-medical places, to detect the DVT of a lower limb. The system included a wearable device with commercial reflective PPG sensors and an accelerometer to measure LRR signals, and a mobile application (APP) to control the works of sensors, guide the rhythm of foot action, and display and store the LRR signals in real time. An array sensor embedded with three reflective PPG sensors was designed to detect the change of venous blood flow during the calf pumping. An accelerometer was placed at the foot to simultaneously monitor the dorsiflexion action at the ankle. In order to verify this prototype, twenty healthy subjects were included to participate in this study. The veins of a lower limb were partially or totally occluded by inflating the cuff up to 100 mmHg or 150 mmHg to simulate the stenosis phenomenon in slight or serious DVT scenarios, respectively. The identifiable rules for determining the quality of the LRR signals from the different PPG sensors were studied. Thus, the LRR signal with the best quality and sensitivity was chosen. Six characteristic parameters regarding the LRR signal were defined as the slopes of emptying and refilling curves in the LRR signal and the change of venous pump volume. Those parameters were used to classify whether the DVT occurrence was positive or negative by a receiver operating characteristic (ROC) curve. The results showed that the prototype of the examination system has the potential for screening for DVT in non-medical environments.

## 2. Materials and Methods

**Figure 1:**
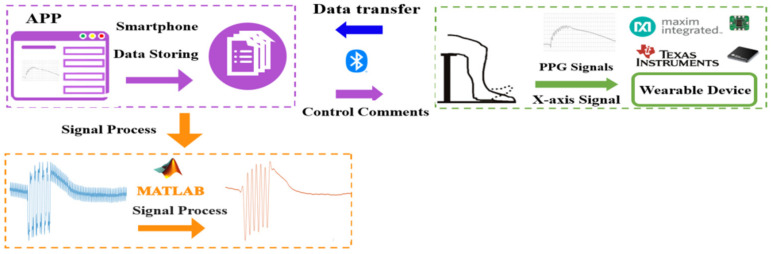
Architecture of the deep vein thrombosis (DVT) examination system. APP is the mobile application and PPG is the photoplethysmography.

Figure 1 shows the architecture of the DVT examination system. The purple block is the APP that can display and store the LRR signals in real time, show the rhythm of dorsiflexion, and control the wearable device. The green block represents the wearable device that uses Bluetooth communication to receive APP comments and send the LRR data to the APP. The reflective PPG sensors and accelerometer are utilized to measure the LRR and the foot action signals, respectively. The orange block denotes the recorded LRR signals and the *x*-axis signal of the accelerometer that are uploaded to a personal computer for further smoothing of the LRR signals and for acquiring the characteristic parameters from the LRR signals with Matlab R2015a version.

### 2.1. LRR Wearable Device

**Figure 2:**
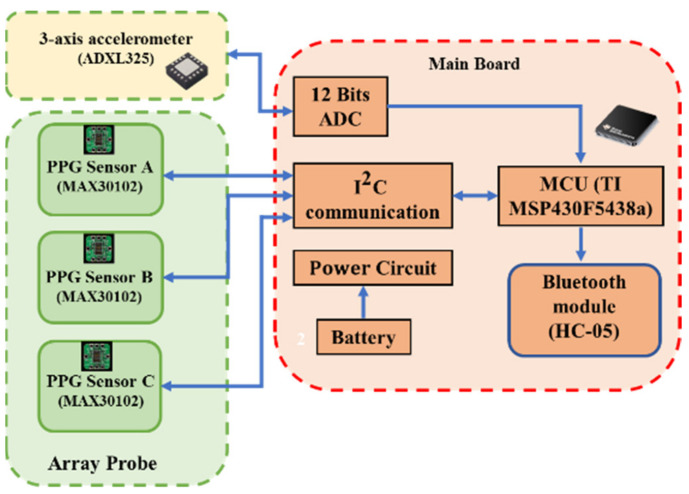
The block diagram of the light reflection rheography (LRR) wearable device consisting of an array-type probe, accelerometer, and main board. PPG is the photoplethysmography, MCU the microcontroller unit. and ADC the analog to digital converter.

**Figure 3:**
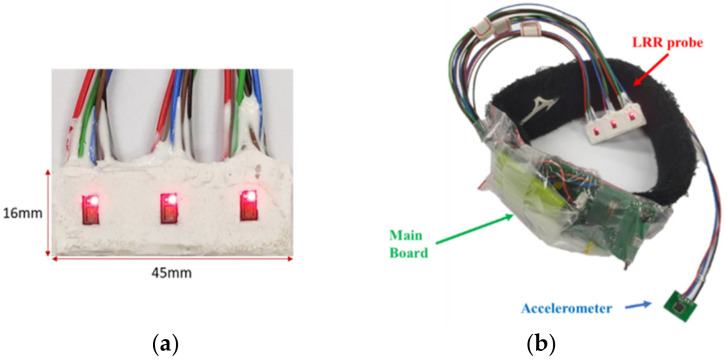
(**a**) Real photo of the array-type probe, (**b**) the real photo of the LRR wearable device.

Figure 2 shows the block diagram of the LRR wearable device that includes an array-type probe, accelerometer, and main board. The array-type probe consists of three MAX30102 PPG sensors (Maxim Integrated TM, San Jose, CA, USA), whose size is 16 mm × 45 mm, as shown in Figure 3a. Each MAX30102 PPG sensor contains two light-emitting diodes (LEDs), with wavelengths of 660 nm (red light) and 880 nm (infrared light). Moreover, a photo diode, an 18-bit analog-to-digital converter, and low-noise electronics with ambient light rejection are also included in this PPG sensor. Communication between the sensors and a microcontroller unit (MCU) is accomplished via a standard I2C-compatible interface. The sampling rate is set to 100 Hz. The *x*-axis signal of the accelerometer (ADXL325, Analog Devices, Norwood, MA, USA) is sampled by the MCU with a 12-bit analog-to-digital converter. The MCU of the main board is a 16-bit microcontroller (MSP430F5438A, Texas Instruments TM, Dallas, TX, USA), which reconciles the workflow of the MAX30102 and ADXL325, performs the signal processing to obtain the DVT-related parameters, and transmits the data to the APP by a Bluetooth module (HC-05, Itead, Shenzhen, China). The power circuit includes a Li battery (150 mAh), a charging integrated circuit (IC) (TIBQ24072), and a regulator IC (XC62FP, 3.3 V). The size of the main board is 54 mm × 70 mm. Figure 3b shows the real picture of the LRR wearable device.

### 2.2. Signal Processing

**Figure 4:**
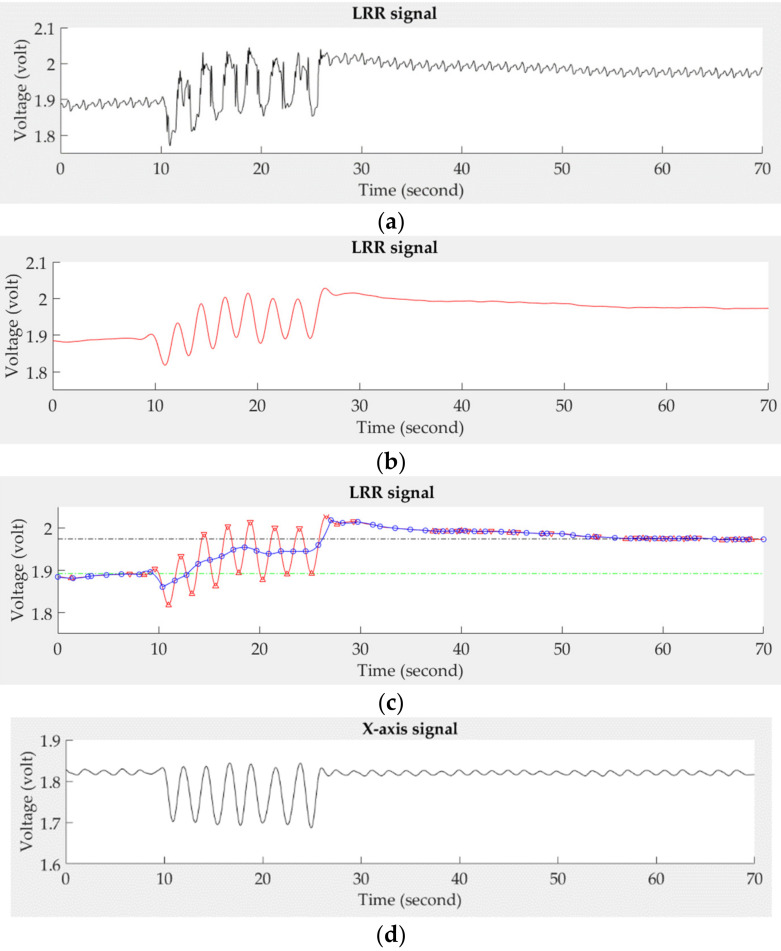
The signal processing steps for a normal LRR signal, (**a**) original signal, (**b**) the filtered signal, (**c**) the smooth (blue line) and filtered (red line) signals, (**d**) the synchronous *x*-axis signal.

The LRR signal measured by PPG sensors is a direct current (DC) component of the PPG signal, representing the change of venous blood flow. However, The PPG signal not only contains the venous flow, but also contains the pulses of arterial blood flow. Thus, the proposed system applied a fourth-order Butterworth low-pass filter with a cut-off frequency of 0.5 Hz to filter out high-frequency noise and used a three-point interpolation technique to smooth the LRR signal. The zero-crossing points of the differential filtered signal were used to find the peaks and valleys of the LRR signal. Then, the median point between the neighbor peak and valley was found in the LRR signal. The continuous three median points were used to build the LRR interpolation signal by a second-order polynomial function for approximating the smooth LRR signal. Figure 4 shows the LRR signal at different steps in the digital signal processing. Figure 4a is the original LRR signal that couples with the arterial pulses and the change of venous blood flow. Figure 4b shows the output of this filter. The next process after filtering is to find the peaks and valleys of the filtered LRR signal (red line) marked by the inverse triangle symbols, followed by differentiation, as shown in Figure 4c. The median points are marked by the circle symbols for building the smooth signal (blue line). Additionally, the synchronous *x*-axis signal is shown in Figure 4d.

### 2.3. Characteristic Parameters of the LRR Signal

**Figure 5:**
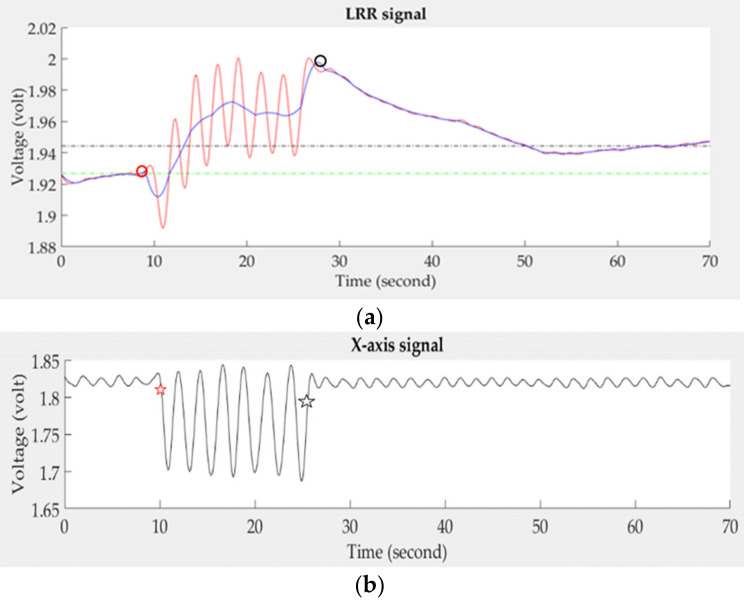
(**a**) Four characteristics of the smooth LRR signal (blue line), LRR_start_ (red circle symbol), LRR_end_ (black circle symbol), resting baseline (short green dash line), and refilling baseline (long blue dash line), (**b**) two characteristics of the accelerometer signal, X_start_ and X_end_ (red and black star symbols).

Figure 5a shows four characteristics in the smooth LRR signal (blue line), the start (red circle symbol), and the end (black circle symbol) points in the emptying phase, and the two baselines (short green dash line and long blue dash line) in the resting and refilling phase. The filtered LRR signal is represented by the red line. In Figure 5b, we find that the *x*-axis signal has larger alternating amplitudes in the emptying phase than those in the resting and refilling phases. Thus, the first maximum negative and last positive changed points of the *x*-axis signal is used to define the start and end points, X_start_ and X_end_ (red and black star symbols), in the emptying phase. Then, the start maximum point of the smooth LRR signal, LRR_start_ (red circle symbol), is found between before and after 50 points of X_start_, and the end maximum point of the smooth LRR signal, LRR_end_ (black circle symbol), is found between before and after 50 points of X_end_, as shown in Figure 5a.

The average of the LRR signal within five seconds before LRR_start_ corresponds to the resting baseline (short green dash line), and the average within the five seconds before the ending measurement represents the refilling baseline (long blue dash line). In the study, six characteristic parameters from the LRR signal were defined to identify the DVT, as follows. VP_1_ is defined as the change of venous pump volume between the LRR_end_ and resting baseline, VP_2_ is the change of venous pump volume between the LRR_end_ and refilling baseline, m_10_ is the slope of the empty curve from the LRR_end_ to LRR_start_, m_40_ is the slope of the refilling curve from the LRR_end_ to the point at 40 s, m_50_ is the slope of the refilling curve from the LRR_end_ to the point at 50 s, and m_60_ is the slope of the refilling curve from the LRR_end_ to the point at 60 s.

### 2.4. Quality of LRR Signals

**Figure 6:**
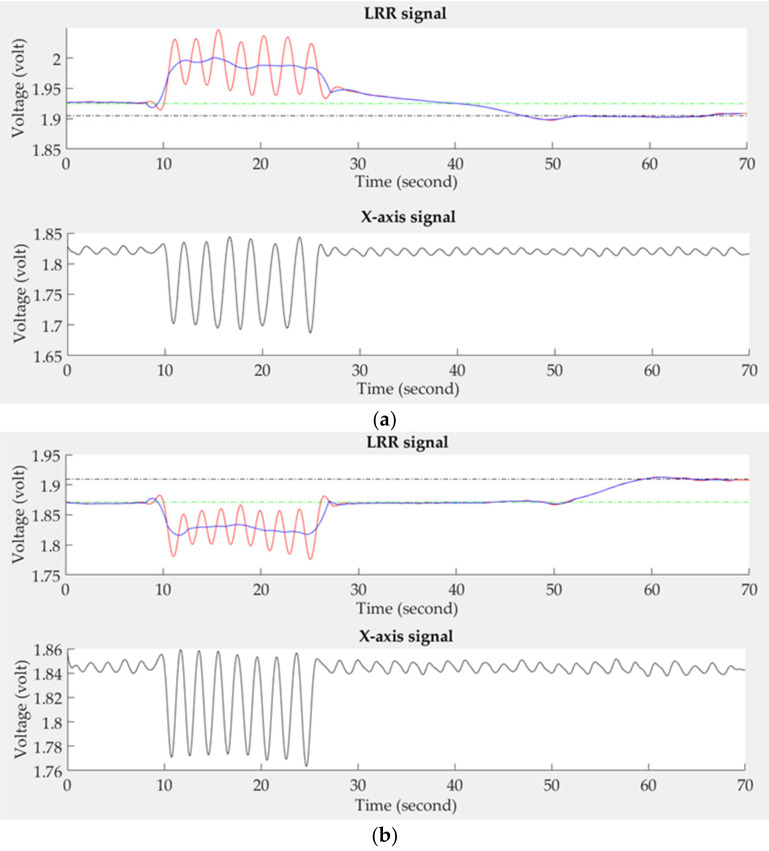
(**a**) The phase angle between the LRR signal and the *x*-axis signal is 180° because the PPG sensor detects the upstream of venous blood flow, (**b**) The LRR signal has the drift phenomenon.

Two fundamental rules for determining the quality of LRR signals on the different PPG sensors were formulated. The first rule states that, according to LRR, the PPG sensor should detect the downstream of venous blood flow in the emptying phase. If the PPG sensor detects the upstream of venous blood flow, the sensor may have been placed at a wrong position. Thus, when the phase angle between the filtered LRR signal (red line) and *x*-axis signal is larger than 90°, such an LRR signal is considered of low quality, as show in Figure 6a. The second rule states that, when the foot is moving to empty the deep venous blood, the PPG sensor may move to a different place. The baseline of the LRR signal has a drift phenomenon. Thus, when VP_1_ or VP_2_ is negative, the LRR signal is determined to be of low quality, as show in Figure 6b.

### 2.5. APP Program

**Figure 7:**
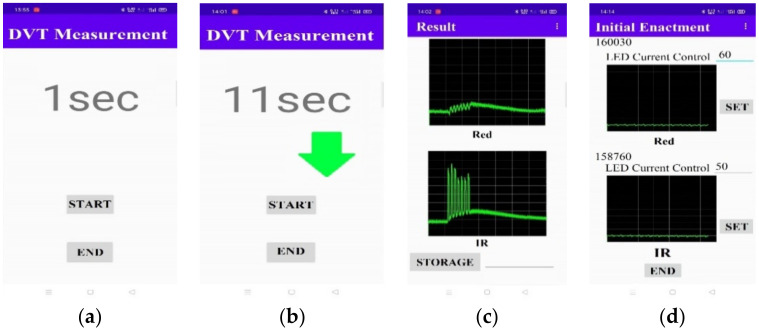
The screen displays of the APP program: (**a**) starting or ending the measurement, (**b**) the rhythm of foot action, (**c**) measured LRR signals, (**d**) adjustment of LED currents.

In the study, an APP program is designed to control the functions of the wearable device (Figure 7a), to instruct the rhythm of foot action (Figure 7b), and to display the results of the LRR signal in real time (Figure 7c). In the beginning, the APP program searches for the Bluetooth (BT) module of the wearable device and automatically connects with it. Users can adjust the LED current to shift the baseline of the LRR curve, as shown in Figure 7d. Then, the APP starts the DVT measurements, as shown in Figure 7a.

### 2.6. Protocol of Experiment

This study recruited twenty healthy subjects (10 male and 10 female) without cardiovascular disease or injured limbs. Their age was between 20 and 24 years (21.8 ± 1.2 years, mean ± standard deviation), weight between 38 and 80 Kg (60.9 ± 10 Kg), and height between 150 and 180 cm (164.4 ± 9.2 cm). The vein of one lower limb was occluded by pressuring the cuff up to 100 mmHg and 150 mmHg to simulate the slight and serious DVT scenarios. This experiment was approved by the Research Ethics Committee of China Medical University & Hospital (No. CMUH107-REC3-061), Taichung, Taiwan.

**Figure 8:**
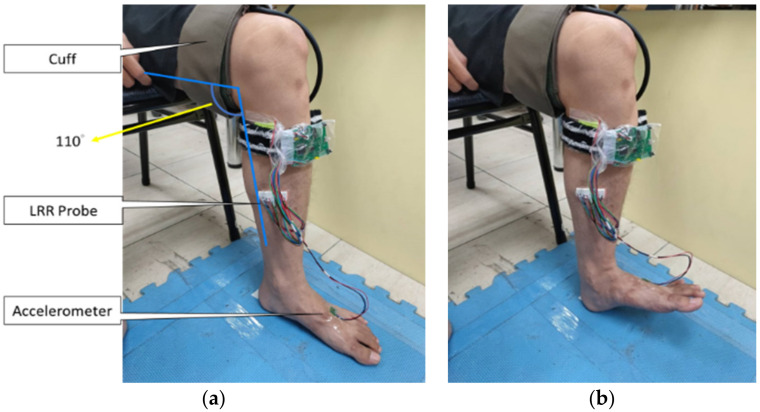
Real photo of LRR measurement: (**a**) placement of the cuff, LRR probe, and accelerometer, (**b**) the foot action.

Subjects were requested to comfortably sit on a chair with the foot resting flat on the floor, and the angle between calf and thigh was about 110°, as show in Figure 8a. A LRR probe was placed at the distal end of the calf muscle, which was fixed by transparent tape. An accelerometer was placed at the front end of the foot and an air cuff was wrapped around the thigh. The APP algorithm connected with the LRR device via Bluetooth. The APP displayed the LRR signal in real time. The light lumen of LED in the reflective PPG sensors affect the signal to noise ratios (SNRs) of the red-light and IR-light photo diodes, and skin color, skin thickness, and soft tissue also affect transmissible light lumen of the LED. To standardize the SNRs of the red-light and IR-light LRR signals for every subject, the baselines of the IR-light LRR signals for three PPG sensors were adjusted to 1.9 volts before measurement. Subjects were requested to move their feet. If the LRR signals had an emptying curve, it meant the LRR probe was placed at the right position. If not, the LRR probe was repositioned until an emptying curve appeared. Subjects were measured three times in the first experiment on the first day. The following week, they were asked to perform the second experiment. In the first measurement, the thigh was not occluded in the negative group. However, in the positive group, the thigh was partially or totally occluded by inflating the cuff up to 100 mmHg or 150 mmHg, respectively, to simulate the stenosis occurrence in mild or severe DVT. Subjects were requested to take a 10-min rest between the two measurements. They could move their feet to augment the venous blood flow. During the first measurement, the resting phase lasted for 10 s, the emptying phase 15 s, and the refilling phase 45 s. When the LRR probe was placed at the right position, users could push the start icon of the APP to record the displayed LRR signals in real time. In the emptying phase, the APP showed the rhythm of the foot action, as shown in Figure 8b. Subjects were requested to move their feet following a rhythm of 0.5 Hz.

In the study, the terms, true positive (TP), false positive (FP), true negative (TN) and false negative (FN), refer to the result of our experiments and the correctness of the classification. TP means that a subject was correctly diagnosed as DVT, FP means “incorrectly diagnosed as DVT”, TN means “correctly diagnosed as non DVT”, and FN means “incorrectly diagnosed as non DVT”. Here, the performance of the proposed method was evaluated using accuracy, (TP+TN)/(TP+FP+FN+TN); precision, TP/(TP+FP); sensitivity, TP/(TP+FN); and specificity, TN/(FN+TN).

## 3. Results

Table 1 shows the numbers of LRR signals with good quality for each subject. In the first experiment, there are no good-quality LRR signals in the red-light measurement under the venous non-occluded and occluded scenarios for Subject 9. For Subjects 8 and 15, there are no good-quality LRR signals under only one scenario. In the second experiment, for Subjects 3, 4, 9, and 11, some LRR signals with good quality are missing under the different venous occluded scenarios. Consequently, the total numbers of LRR signals with good quality in the red-light and IR-light measurements both are 224.

**Figure 9:**
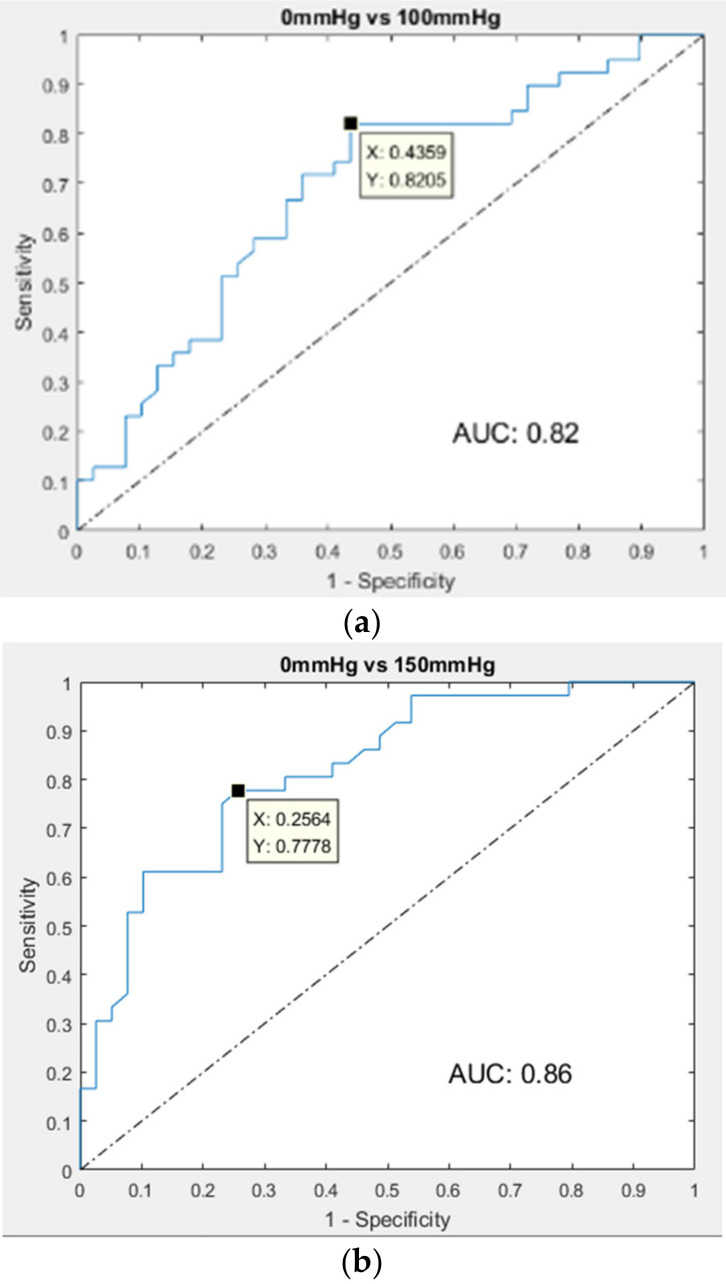
ROC curves of m_40_: (**a**) the area under the curve is 0.82 in the slight DVT scenario, (**b**) the area under the curve is 0.86 in the serious DVT scenario.

Table 2 shows the statistical analyses of six characteristic parameters under the different venous occluded scenarios in the red-light and IR-light measurements. We used the *t*-test to assess the performances of six parameters for the sensitivity of DVT detection. A *p*-value of 0.05 or lower is considered statistically significant (represented in red) between the negative (0 mmHg) and positive (100 and 150 mmHg) groups. We find that all parameters have significant differences between the negative and positive groups. However, under the same scenario comparison (0 mmHg vs. 150 mmHg), *p*-values of the six parameters in the IR-light measurement are lower than those in the red-light measurement. Moreover, the absolute mean values of six parameters all decrease as the colluded pressure increases. To select the best sensitivity value of each parameter from three LRR signals, we analyzed the maximum, median, and minimum values of parameters under the different venous occluded scenarios in the red-light and IR-light measurements, as shown in Table 3. If there were two LRR signals with good quality, the median value was the arithmetic average. If there was one LRR signal with good quality, maximum, median, and minimum values were the same values. One of three values for each parameter had the lowest *p*-value, which was the optimal value to detect DVT. We find that m_10_, VP_1_, and VP_2_ should select the maximum values; m_50_ and m_60_ should select the minimum values; and m_40_ should select the median values. An ROC curve was used to evaluate the performances of six parameters in the IR-light measurement in the various DVT scenarios. Table 4 shows the fusion matrix and performances of the six parameters. The first three parameters with better accuracy are m_10_, m_40_, and m_50_ in the slight DVT scenario (0 mmHg vs. 100 mmHg), having accuracies of 72%, 69%, and 69%, respectively. The first three parameters in the serious DVT scenario (0 mmHg vs. 150 mmHg) are m_40_, m_50_, and m_10_, with accuracies of 76%, 73%, and 73%, respectively. The first three parameters with a larger ROC area in the slight DVT scenario are m_40_, m_10_, and VP_2_, with areas of 0.82, 0.79, and 0.78, respectively. The first three parameters with larger ROC area in the serious DVT scenario are m_40_, VP_2_, and m_50_, with areas of 0.86, 0.81, and 0.81, respectively. Therefore, m_40_ and m_10_ have the best performances to detect the DVT conditions in the serious DVT scenario, meaning that the cut points are −1.79 mV/s and 3.05 mV/s. Figure 9a shows the ROC curve of m_40_ in the slight DVT scenarios, and Figure 9b shows the ROC curve in the serious DVT scenario. When its sensitivity is 100% in the serious DVT scenario, its corresponding specificity is 20.2%.

## 4. Discussion

DVT is a prevalent disease with an occurrence of between 2.5% to 5% in the total population, and about 30−40% of DVT patients will finally suffer from pulmonary embolism [26]. The diagnosis of DVT in the clinic is notoriously unreliable because only about 30% of patients have been shown to be positive on objective testing [6,27]. Therefore, suspected DVT in clinical diagnosis could lead to the unnecessary hospitalization of patients, and inappropriate anticoagulation therapy with potentially dangerous consequences.

The LRR technique has been used in several studies for the noninvasive examination of patients with suspected lower limb DVT [28,29]. The technique uses the parameters of the LRR curve, such as the amplitude and rate of venous emptying [30], the time of venous refilling [28], and the shape of the LRR curve [16]. Tan et al. proposed using the amplitude of venous emptying and time of venous refilling to test DVT diagnosis and found that the two parameters achieved an optimal sensitivity of 100% and 100%, and specificities of 35% and 47%, respectively. Additionally, the areas under the ROC curves for the two parameters are 0.74 and 0.75, respectively [29]. In the present study, one of our findings is that the performance of the IR-light measurement is better than that of the R-light measurement (Table 2 and Table 3). The reason for this is partially due to deeper penetrating tissue by the IR light than by the red light [31]. Thus, the LRR signal with the IR-light measurement has a higher sensitivity for the positive DVT group. The accuracies and ROC areas of m_40_ and m_10_ in the serious DVT scenario are 76% and 73%, and 0.86 and 0.78, respectively. Moreover, for various venous occluded scenarios, the performance of m_40_ is significantly lower using the occluded pressure of 100 mmHg than the occluded pressure of 150 mmHg, with sensitivity and specificity of 100% and 10% vs. sensitivity and specificity of 100% and 20.2%, respectively. The present results seem no better than previous ones [14,15,16]. The main reason for this could be that our study does not examine real DVT patients. In the present study, embolization due to thrombus is simulated by directly occluding veins through a high pressure (100 mmHg or 150 mmHg) cuff wrapping around the thigh. The cuffs inflated up to 100 and 150 mmHg are employed to simulate the mild or serious stenosis in deep veins. The deep veins of lower limbs in the 20 subjects may not effectively respond to the phenomenon of embolization in real time. It is found that the results for DVT detection are always better under the 150 mmHg compared with the 100 mmHg. Thus, such finding may, in part, explain this problem.

The study has successfully developed a DVT examination system that may be available in non-medical places and help to examine whether users have DVT of lower limbs. To do so, an obvious challenge is how to obtain an optimal position for the LRR probe in order to apply the system. In the first rule proposed in the study, both the accelerometer and the LRR signals in the emptying phase are used to determine if the PPG sensor detects the upstream or downstream venous blood flow. Once the LRR curve is obtained from the downstream of the venous blood flow, it can be further utilized to find two characteristic parameters, m_40_ and m_10_. In the second rule, the values of both VP_1_ and VP_2_ parameters should be greater than zero. If the value of either the VP_1_ or the VP_2_ parameter is negative, the baseline drift phenomenon must exist in the LRR curve operation.

This study aimed to develop the prototype of an examination system used at non-medical places for detecting DVT in lower limbs using LRR. The prototype of this DVT examination system shows that the commercial reflective PPG sensors and accelerometer could be used for deep vein thrombosis (DVT) detection in lower limbs at a non-medical place. Moreover, based on a wireless technique, this measurement system can be operated to control the actions of sensors and display and store the LRR signals on the APP of a smartphone. To comply with the Subject Consent Form in the approved Institutional Review Board (IRB) project, the authors were only allowed to recruit healthy subject to do the related clinical trials. The DVT with stenosis phenomenon in unhealthy participants was reasonably simulated by inflating the cuff up to 100 mmHg or 150 mmHg. Although two characteristic parameters, m40 and m10, in the PPG signals measured with the prototype are available for distinguishing the negative from the positive group, we did not prove that these two parameters would become standard parameters for the DVT examination. The reason for this is that the recruited subjects were all healthy adults. In the next study, the authors will again apply the IRB for clinical trials including healthy and unhealthy subjects based on the current findings in applying the prototype.

In the system, an array of optical sensors and a motion sensor were designed. Users are asked to only put the two kinds of sensors at the proper places, and then operate the APP. The APP will automatically connect the wearable device to a smartphone and test the LRR signals, and the users should follow the instruction of the APP to move their foot. Finally, the examination reports will be promptly displayed by means of the APP. Moreover, the approved IRB only allows us to perform the clinical trial on healthy subjects. Thus, the included subjects all were the healthy young subjects (50% male and 50% female). However, based on the current results in this study for DVT detection, the authors will apply the IRB for the clinical trial on patients with DVT in the next study.

## 5. Conclusions

The wearable device must be user-friendly and easy to operate, and it must be usable at non-medical institutes. Based on the LRR, the examination system has been developed for screening tests in subjects with lower limb DVT and holds several advantages over previous LRR instruments. The proposed system includes a wearable device and APP program. The wearable device not only has an array probe with three reflective PPG sensors, but also has an accelerometer to detect the foot action conditions. The wearable device can compensate blood displacement for different cutaneous optical densities because of skin color, skin thickness, and soft tissue, and it permits quantitative evaluation of vein position by the quality of the LRR signal. The APP program can control the functions of the wearable device, instruct the rhythm of the foot action, and display and store the LRR signals. Twenty healthy subjects were recruited to perform experiments to verify the performance of this prototype. One important finding is that the two characteristic parameters, m40 and m10, both can be applied to detect the DVT of loer limbs without missing any positive cases. This suggests that the prototype of the examination system has the potential for screening for DVT in non-medical environments.

## Figures and Tables

**Table 1:** The numbers of LRR signals with good quality for each subject.

Subject No.	First Experiment						Second Experiment						Total	
	R-Light			IR-Light			R-Light			IR-Light			R-Light	IR-Light
	0	100	150	0	100	150	0	100	150	0	100	150		
1	2	3	3	2	2	2	2	1	3	1	1	2	14	10
2	2	0	2	2	1	1	2	2	1	1	1	1	9	7
3	2	2	2	2	2	2	1	1	2	0	1	0	10	7
4	1	2	1	2	2	2	1	0	0	1	1	0	5	8
5	1	2	3	2	2	2	2	2	2	2	2	2	12	12
6	1	2	2	1	1	2	3	3	3	3	3	3	14	13
7	1	1	2	2	1	1	1	1	2	1	2	2	8	9
8	2	2	2	2	0	2	1	1	1	1	1	1	9	7
9	0	0	0	2	1	1	1	0	0	1	1	0	1	6
10	2	2	3	2	3	1	2	1	2	2	2	2	12	12
11	1	3	3	2	2	3	1	1	0	2	2	1	9	12
12	3	3	3	3	3	3	2	2	2	2	1	2	15	14
13	2	3	3	2	2	2	3	3	3	3	3	2	17	14
14	1	1	1	1	2	1	2	2	2	2	2	2	9	10
15	3	1	2	3	2	0	1	1	1	2	2	2	9	11
16	3	3	3	3	3	3	3	3	3	3	3	2	18	17
17	3	3	3	3	3	3	2	2	2	1	1	2	15	13
18	1	2	2	1	1	3	2	2	1	3	3	2	10	13
19	3	1	1	3	2	2	3	3	3	3	3	3	14	16
20	3	3	2	3	2	2	2	2	2	2	2	2	14	13
Sum	37	39	43	43	37	38	37	33	35	36	37	33	224	224

**Table 2:** Statistical analyses of six parameters between the negative group (0 mmHg) and positive groups (100 mmHg and 150 mmHg) by *t*-test. A *p*-value of less than 0.05 is highlighted in red.

	R-Light			IR-Light		
	0 mmHg	100 mmHg	150 mmHg	0 mmHg	100 mmHg	150 mmHg
	(N = 74)	(N = 72)	(N = 78)	(N = 79)	(N = 74)	(N = 71)
m_10_	4.76 ± 2.47	3.86 ± 2.53	3.74 ± 2.40	3.41 ± 1.96	2.54 ± 1.84	2.11 ± 1.48
(mV/s)		(0.0325)	(0.0108)		(0.0056)	(1.2 × 10^−5^)
m_50_	−1.99 ± 1.26	−1.28 ± 0.89	−1.21 ± 0.81	−1.86 ± 1.20	−1.28 ± 0.92	−0.89 ± 0.58
(mV/s)		(0.0002)	(1.1 × 10^−5^)		(0.0010)	(4.1 × 10^−9^)
VP_1_	73.7 ± 37.7	60.2 ± 39.2	58.7 ± 38.0	54.3 ± 31.4	39.8 ± 29.0	33.2 ± 23.5
(mV)		(0.0365)	(0.0158)		(0.0037)	(7.6 × 10^−6^)
VP_2_	53.0 ± 30.8	38.0 ± 24.1	40.0 ± 23.0	47.2 ± 29.0	33.4 ± 23.8	24.5 ± 14.8
(mV)		(0.0013)	(0.0035)		(0.0017)	(1.7 × 10^−8^)
m_40_	−2.67 ± 2.06	−1.67 ± 1.25	−1.30 ± 1.07	−2.74 ± 1.86	−1.96 ± 1.31	−1.30 ± 0.91
(mV/s)		(0.0007)	(8.0 × 10^−7^)		(0.0032)	(1.6 × 10^−8^)
m_60_	−1.55 ± 0.92	−1.07 ± 0.70	−1.12 ± 0.67	−1.41 ± 0.88	−0.98 ± 0.70	−0.70 ± 0.44
(mV/s)		(0.0006)	(0.001)		(0.0012)	(8.2 × 10^−9^)

**Table 3:** Statistical analyses of six parameters, being maximum, median, and minimum values between the negative group (0 mmHg) and positive groups (100 mmHg and 150 mmHg) by *t*-test. A *p*-value of less than 0.05 is highlighted in red.

		R-Light			IR-Light		
		0 mmHg	100 mmHg	150 mmHg	0 mmHg	100 mmHg	150 mmHg
VP_1_ (mV)	Maxi.	87.4 ± 39.8	74.5 ± 46.2	71.5 ± 43.1	65.5 ± 32.6	48.5 ± 34.9	40.6 ± 25.7
			(0.1969)	(0.0997)		(0.0284)	(0.0005)
	Med.	74.9 ± 35.0	65.0 ± 43.6	58.8 ± 36.3	54.7 ± 26.8	42.5 ± 34.1	34.3 ± 23.0
			(0.2819)	(0.0556)		(0.0840)	(0.0007)
	Min.	62.0 ± 35.4	55.8 ± 44.5	46.4 ± 32.0	43.1 ± 26.1	36.7 ± 34.6	28.2 ± 22.6
			(0.5029)	(0.0501)		(0.3618)	(0.0104)
VP_2_ (mV)	Maxi.	65.4 ± 33.4	48.7 ± 26.0	47.7 ± 25.5	58.4 ± 31.6	39.4 ± 26.0	30.4 ± 15.2
			(0.0192)	(0.0125)		(0.0049)	(7.2 × 10^−6^)
	Med.	54.7 ± 29.2	40.6 ± 23.2	40.3 ± 21.2	48.4 ± 26.6	34.1 ± 23.8	25.2 ± 12.9
			(0.0244)	(0.0182)		(0.0145)	(1.0 × 10^−5^)
	Min.	44.2 ± 30.4	33.0 ± 24.0	33.7 ± 19.3	38.1 ± 26.3	28.8 ± 24.0	20.7 ± 13.2
			(0.0839)	(0.0820)		(0.1056)	(0.0006)
m_10_ (mV/s)	Maxi.	5.65 ± 2.60	4.83 ± 2.97	4.64 ± 2.64	4.15 ± 2.03	3.07 ± 2.19	2.59 ± 1.57
			(0.2098)	(0.1019)		(0.0263)	(0.0004)
	Med.	4.84 ± 2.24	4.21 ± 2.76	3.79 ± 2.25	3.42 ± 1.64	2.72 ± 2.16	2.19 ± 1.42
			(0.2800)	(0.0466)		(0.1119)	(0.0192)
	Min.	3.99 ± 2.23	3.59 ± 2.81	2.98 ± 2.04	2.64 ± 1.59	2.37 ± 2.23	1.80 ± 1.42
			(0.4841)	(0.0452)		(0.5433)	(0.0192)
m_40_ (mV/s)	Maxi.	−2.10 ± 2.00	−1.21 ± 1.01	−0.93 ± 0.91	−2.11 ± 1.66	−1.53 ± 1.08	−1.02 ± 0.69
			(0.0196)	(0.0021)		(0.0723)	(0.0004)
	Med.	−2.83 ± 1.99	−1.74 ± 0.96	−1.36 ± 0.91	−2.83 ± 1.72	−1.90 ± 1.14	−1.33 ± 0.77
			(0.0036)	(0.0001)		(0.0069)	(8.6 × 10^−6^)
	Min.	−3.48 ± 2.29	−2.30 ± 1.32	−1.85 ± 1.12	−3.46 ± 2.08	−2.30 ± 1.41	−1.66 ± 0.98
			(0.0079)	(0.0002)		(0.0051)	(1.1 × 10^−5^)
m_50_ (mV/s)	Maxi.	−1.62 ± 1.22	−1.07 ± 0.86	−0.97 ± 0.64	−1.47 ± 1.04	−1.06 ± 0.86	−0.72 ± 0.48
			(0.0254)	(0.0048)		(0.0597)	(0.0002)
	Med.	−2.06 ± 1.17	−1.37 ± 0.82	−1.23 ± 0.70	−1.91 ± 1.11	−1.28 ± 0.87	−0.92 ± 0.49
			(0.0044)	(0.0005)		(0.0074)	(4.1 × 10^−6^)
	Min.	−2.48 ± 1.35	−1.69 ± 0.96	−1.56 ± 0.89	−1.06 ± 1.33	−1.52 ± 1.01	−1.13 ± 0.60
			(0.0047)	(0.0009)		(0.0041)	(5.2 × 10^−6^)
m_60_ (mV/s)	Maxi.	−1.30 ± 0.91	−0.92 ± 0.70	−0.94 ± 0.55	−1.13 ± 0.81	−0.83 ± 0.70	−0.59 ± 0.38
			(0.0536)	(0.048)		(0.084)	(0.0005)
	Med.	−1.61 ± 0.87	−1.14 ± 0.68	−1.13 ± 0.60	−1.43 ± 0.82	−0.99 ± 0.70	−0.73 ± 0.38
			(0.0132)	(0.0083)		(0.0108)	(7.9 × 10^−6^)
	Min.	−1.92 ± 0.99	−1.40 ± 0.77	−1.35 ± 0.74	−1.74 ± 0.96	−1.16 ± 0.78	−0.88 ± 0.45
			(0.0112)	(0.0061)		(0.0039)	(5.5 × 10^−6^)

**Table 4:** The fusion matrix, performances, cut points, and receiver operating characteristic (ROC) areas of six parameters. TP is the true positive, TN the true negative, FP the false positive, and FN the false negative.

Parameter	DVT Scenario	TP	TN	FP	FN	Acc. (%)	Pre. (%)	Sen. (%)	Spe. (%)	Cut Point	ROC Area(%)
VP_1_ (mV)	100 mmHg	26	27	12	13	0.68	0.68	0.67	0.69	45	0.76
	150 mmHg	20	35	4	16	0.73	0.83	0.56	0.9	31	0.76
VP_2_ (mV)	100 mmHg	29	23	16	10	0.67	0.64	0.74	0.59	41	0.78
	150 mmHg	19	35	4	17	0.72	0.83	0.53	0.9	26	0.81
m_10_ (mV/s)	100 mmHg	29	27	12	10	0.72	0.71	0.74	0.69	3.15	0.79
	150 mmHg	28	27	12	8	0.73	0.7	0.78	0.69	3.05	0.78
m_40_ (mV/s)	100 mmHg	32	22	17	7	0.69	0.65	0.82	0.56	−2.31	0.82
	150 mmHg	28	29	10	8	0.76	0.74	0.78	0.74	−1.79	0.86
m_50_ (mV/s)	100 mmHg	31	23	16	8	0.69	0.66	0.79	0.59	−1.76	0.76
	150 mmHg	26	29	10	10	0.73	0.72	0.72	0.74	−1.35	0.81
m_60_ (mV/s)	100 mHg	30	23	16	9	0.68	0.65	0.77	0.59	−1.33	0.76
	150 mmHg	22	32	7	14	0.72	0.76	0.61	0.82	−0.88	0.80

Note: Acc., Pre., Sen., and Spe. are abbreviations for accuracy, precision, sensitivity, and specificity, respectively.

## Data Availability

Requests for data should be addressed to S.-H.L.

## References

[1] Jaff M.R., McMurtry M.S., Archer S.L., Cushman M., Goldenberg N., Goldhaber S.Z., Jenkins J.S., Kline J.A., Michaels A.D., Thistlethwaite P. (2011). Management of massive and submassive pulmonary embolism, iliofemoral deep vein thrombosis, and chronic thromboembolic pulmonary hypertension. Circulation.

[2] Lee C.H., Lin L.J., Cheng C.L., Kao Yang Y.H., Chen J.Y., Tsai L.M. (2010). Incidence and cumulative recurrence rates of venous thromboembolism in the Taiwanese population. J. Thromb. Haemost..

[3] Ansari M.T., Cheung B.M., Qing Huang J., Eklof B., Karlberg J.P. (2005). Traveler’s thrombosis: A systematic review. J. Travel Med..

[4] Scurr J.H., Machin S.J., Bailey-King S., Mackie I.J., McDonald S., Smith P.D. (2001). Frequency and prevention of symptomless deep-vein thrombosis in long-haul flights: A randomised trial. Lancet.

[5] White R.H., McGahan J.P., Daschbach M.M., Hartling R.P. (1989). Diagnosis of deep-vein thrombosis using duplex ultrasound. Ann. Intern. Med..

[6] Lensing A.W., Büller H., Prandoni P., Batchelor D., Molenaar A.H., Cogo A., Vigo M., Huisman P.M., ten Cate J.W. (1992). Contrast venography, the gold standard for the diagnosis of deep-vein thrombosis: Improvement in observer agreement. Thromb. Haemost..

[7] Hull R., Aken W.G., Hirsh J., Gallus A., Hoicka G., Turpie A.G., Walker I., Gent M. (1976). Impedance plethysmography using the occlusive cuff technique in the diagnostic of venous thrombosis. Circulation.

[8] Hull R., Taylor D.W., Hirsh J., Sackett D.L., Powers P., Turpie A.G.G., Walker I. (1978). Impedance plethysmography: The relationship between venous filling and sensitivity and specificity for proximal vein thrombosis. Circulation.

[9] Sandler D.A., Duncan J.S., Ward P. (1984). Diagnosis of deep-vein thrombosis: Comparison of clinical evaluation, ultrasound, plethysmography, and venoscan with X-ray venogram. Lancet.

[10] Ginsberg J.S., Kearon C., Douketis J., Turpie A.G., Brill-Edwards P., Stevens P., Panju A., Patel A., Crowther M., Andrew M. (1997). The use of D-Dimer testing and impedance plethysmographic examination in patients with clinical indications of deep vein thrombosis. Arch. Intern. Med..

[11] Lensing A.W.A., Prandoni P., Brandjes D. (1989). Detection of deep-vein thrombosis by real-time B-mode ultrasonography. N. Engl. J. Med..

[12] Goodacre S., Sampson F., Stevenson M., Wailoo A., Sutton A., Thomas S., Locker T., Ryan A. (2006). Measurement of the clinical and cost-effectiveness of non-invasive diagnostic testing strategies for deep vein thrombosis. Health Technol. Assess..

[13] Shepard A.D., Mackey W.C., O’Donnell T.F., Heggerick P.A. (1984). Light reflection rheography: A new non-invasive test of venous function. Bruit.

[14] Neumann H.A., Boersma I.D.S. (1992). Light reflection rheography: A non-Invasive diagnostic tool for screening for venous disease. J. Dermatol. Surg. Oncol..

[15] Arora S., Lam D., Kennedy C., Meier G.H., Gusberg R.J., Negus D. (1993). Light reflection rheography: A simple noninvasive screening test for deep vein thrombosis. J. Vasc. Surg..

[16] Thomas P.R.S., Butler C.M., Bowman J., Bowman J., Grieve N.W.T., Bennett C.E., Taylor R.S., Thomas M.H. (1991). Light reflection rheography: An effective non-invasive technique for screening patients with suspected deep venous thrombosis. Br. J. Surg..

[17] Prawiro A.P.J., Chou N.-K., Lee M.-W., Lin Y.-H. (2019). A wearable system that detects posture and heart rate: Designing an integrated device with multi-parameter measurements for better health care. IEEE Consum. Electron. Mag..

[18] Yu L., Chan W., Zhao Y., Tsui K.-L. (2018). Personalized health monitoring system of elderly wellness at the community level in Hong Kong. IEEE Access.

[19] Liu S.-H., Wang J.-J., Su C.-H., Tan T.-H. (2018). Development of a patch-type electrocardiographic monitor for real time heartbeat detection and heart rate variability analysis. J. Med. Biol. Eng..

[20] Liu S.-H., Lin C.-B., Chen Y., Chen W., Huang T.-S., Hsu C.-Y. (2019). An EMG patch for the real-time monitoring of muscle-fatigue conditions during exercise. Sensors.

[21] Ijaz M.F., Attique M., Son Y. (2020). Data-driven cervical cancer prediction model with outlier detection and over-sampling methods. Sensors.

[22] Alia F., El-Sappagh S., Islamd S.M.R., Kwake D., Alif A., Imrang M., Kwakh K.-S. (2020). A smart healthcare monitoring system for heart disease prediction based on ensemble deep learning and feature fusion. Inf. Fusion.

[23] Analog Devices. AD8232. http://www.analog.com/en/products/amplifiers/instrumentation-amplifiers/ad8232.html#product-overview.

[24] MAX30101 Datasheet and Product Info Maxim Integrated. https://www.maximintegrated.com/en/products/sensors/MAX30101.html.

[25] Analog Devices. ADXL325. http://www.analog.com/en/products/sensors-mems/accelerometers/adxl325.html.

[26] Geerts W.H., Heit J.A., Clagett G.P., Pineo G.F., Colwell C.W., Anderson F.A., Wheeler H.B. (2001). Prevention of venous thromboembolism. Chest.

[27] Salcuni M., Fiorentino P., Pedicelli A., Di Stasi C. (1996). Diagnostic imaging in deep vein thrombosis of the limbs. Rays.

[28] Abbott G.T., Diggory R.T., Harris I. (1995). Comparison of light reflection rheography with ascending venography in the diagnosis of lower limb deep vein thrombosis. Br. J. Radiol..

[29] Tan Y.K., da Silva A.F. (1999). Digital photoplethysmography in the diagnosis of suspected lower limb DVT: Is it useful?. Eur. J. Vasc. Endovasc. Surg..

[30] Mitrani A.A., Gonzalez M.L., O’Connell M.T., Guerra J., Harwood R.B., Gardner L.B. (1991). Detection of clinically suspected deep vein thrombosis using light reflection rheography. Am. J. Surg..

[31] Liu J., Yan B.P., Zhang Y.T., Ding X.R., Su P., Zhao N. (2019). Multi-wavelength photoplethysmography enabling continuous blood pressure measurement with compact wearable electronics. IEEE Trans. Biomed. Eng..

